# Neurocysticercotic Calcifications and Hippocampal Sclerosis: A Case-Control Study

**DOI:** 10.1371/journal.pone.0131180

**Published:** 2015-07-01

**Authors:** Mateus de Oliveira Taveira, Marcia Elisabete Morita, Clarissa Lin Yasuda, Ana Carolina Coan, Rodrigo Secolin, Alberto Luiz Cunha da Costa, Fernando Cendes

**Affiliations:** 1 School of Medical Sciences, University of Campinas-UNICAMP, Campinas, SP, Brazil; 2 Department of Neurology, University of Campinas—UNICAMP, Campinas, SP, Brazil; 3 Department of Medical Genetics, University of Campinas—UNICAMP, Campinas, SP, Brazil; Sainte-Anne Hospital Center, FRANCE

## Abstract

**Objective:**

The exact role of calcified neurocysticercotic lesions (CNLs) in epilepsy is yet unknown and controversial. Although the relationship between CNLs, epilepsy and mesial temporal lobe epilepsy with hippocampal sclerosis (MTLE-HS) has already been addressed, to our knowledge, no study has actually provided strong statistical evidence, nor reported the ODDS ratio for these associations. Therefore, we designed this case-control study to assess the likelihood of having MTLE-HS versus other forms of epilepsy in the presence of CNLs.

**Methods:**

In this case-control study we included 119 consecutive patients with epilepsy and 106 disease controls (headache) with previous CT scans. We subdivided cases into MTLE-HS and other epilepsies. We used brain CT scans to define presence or absence of CNLs. After exploratory analyses, we used logistic regression to analyze the association between CNLs, epilepsy subgroups and disease controls.

**Results:**

CNLs were found in 31.09% of cases and in 11.32% of controls (p<0.001). The initial analysis comparing epilepsy versus controls revealed a significant association between CNLs and epilepsy (OR = 5.32; 95%CI = 2.43-11.54; p<0.001). However, when we compared MTLE-HS versus other epilepsies versus controls we confirmed that CNLs were associated with MTLE-HS (OR = 11.27, 95%CI = 4.73-26.85; p<0.001) but other epilepsies were not. We found no difference in the CNLs load and no difference in the location of the CNLs when we compared patients with MTLE-HS, other epilepsies and disease controls.

**Significance:**

The inclusion of controls allowed us to estimate the likelihood of having epilepsy in the presence of CNLs. We found that patients with CNLs were 11 times more likely to have MTLE-HS; however, the presence of CNLs did not change the odds of having other types of epilepsy. These findings raise the possibility of neurocysticercosis playing a role in the pathophysiology of MTLE-HS and need further confirmation in other series.

## Introduction

Neurocysticercosis (NCC) infection is considered to be an important cause of epilepsy in developing countries [1, 2] and some studies suggest that up to a third of cases of epilepsy in endemic areas may be attributed to NCC [3–5]. Although the relationship between acute symptomatic seizures and NCC during its transitional stage has long been known [6], the exact role of calcified neurocysticercotic lesions (CNLs) in epilepsy pathogenesis (with or without hippocampal sclerosis (HS)) is yet unclear and controversial. Some studies suggest that there is a clear association between CNLs and epilepsy in general [2,7,8]. Others indicate that CNLs are not as inactive as previously thought, as indicated by perilesional edema, gliosis and contrast enhancement seen in MRI and perfusion MRI studies. [1,9]. However, a previous study showed that the presence of CNLs was not associate with the clinical and pathological findings nor with postsurgical outcomes in patients with HS; thus suggesting that CNLs could represent a coincidental pathology in the context of patients with HS and refractory seizures [10].

Mesial temporal lobe epilepsy (MTLE) is the most common form of epilepsy and HS is its most common pathological substrate [11, 12]. The association between MTLE with HS (MTLE-HS) and CNLs was also suggested by case reports and cross sectional analysis [2, 13, 14]. Some studies even suggest a causative role of CNLs in MTLE-HS [13, 15].

Although the relationship between CNLs, epilepsy and HS has been investigated by many authors [2, 9, 15], to our knowledge, none provided strong statistical evidence, nor reported the ODDS ratio for these associations. Given the controversy and lack of strong statistical evidences, we designed a case-control study to assess the likelihood of MTLE-HS in the presence of CNLs.

## Materials and Methods

### Ethics Statement

Our Research Ethics Committee (Comitê de Ética e Pesquisa- FCM/ Unicamp) approved the study. We were exempted from having to obtain written informed consent from subjects due to the retrospective nature of our study.

### Participants

**Fig 1:**
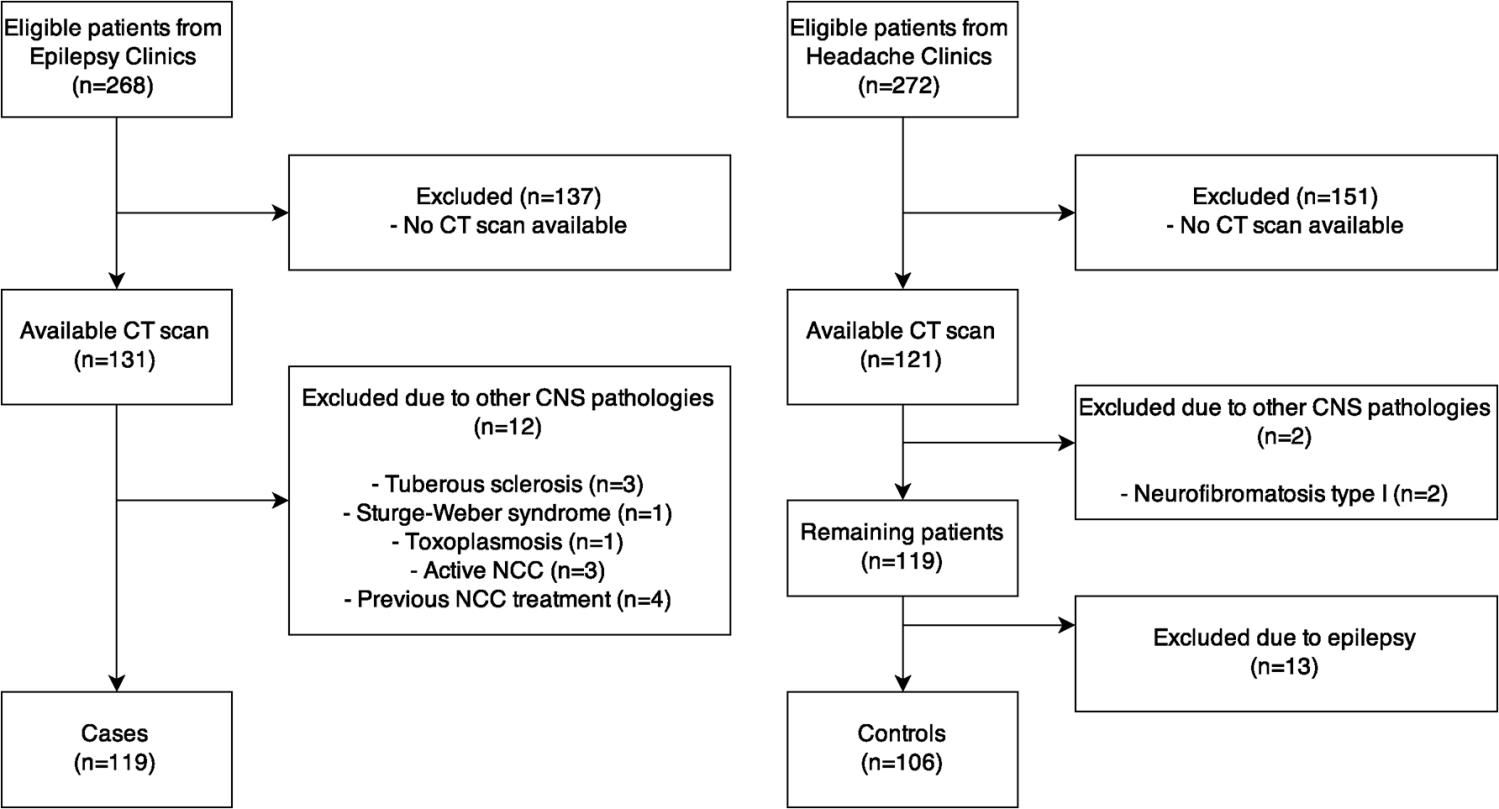
Flow diagram of enrolled cases and controls. Flow diagram showing on the left side how cases were selected and on right side how controls were selected. As can be seen, the number of eligible patients that were excluded due to not having an available CT scan was the largest exclusion criteria in both groups. CT = brain computed tomography scan, CNS = central nervous system, NCC = neurocysticercosis.

We conducted this case-control study at University of Campinas Hospital (HC-UNICAMP). We analyzed data from 268 consecutive cases (with confirmed diagnosis of epilepsy [16]) from our Epilepsy Clinics and 272 disease controls, who attended our Headache Clinics from July 2013 to September 2013 and excluded 315 that did not fulfill the study criteria (Recruitment details are displayed in Fig 1). We included patients who attended our Headache Clinics as “disease controls” (n = 106) and defined patients from our Epilepsy Clinics as “cases” (n = 119). Since we included consecutive individuals, we did not match cases and controls for gender and age. The rationale for choosing Headache Clinics patients as controls was the need to have subjects without epilepsy from the same population, with similar socioeconomic background and who had already been submitted to a brain computed tomography (CT) scan. In addition, most of these patients had migraine or tension type headache, and even if the CNLs were somewhat related to the headache, they did not have epilepsy.

Inclusion criteria for both groups were having a brain CT scan and being older than 15 years old. The rationale for choosing this imaging method was because CT is superior to MRI scan to detect small calcified lesions [17]. However, all cases had also been submitted previously to a high resolution MRI with an epilepsy protocol for investigation of any underlying epileptogenic lesion in our Epilepsy Clinics.

Exclusion criteria for both groups were having progressive central nervous system (CNS) disease, CNS inflammatory disease, other diseases that may present with CNS calcifications such as neurotuberculosis and neurotoxoplasmosis, CT or MRI lesions characteristic of active NCC and patients with history of previously treated NCC. Patients with suspected CNS inflammatory disease at any point of their history, with inflammatory changes in the cerebral spinal fluid or other positive immunological tests, as well as those with CT or MRI suggesting any acute inflammatory lesions (i.e. contrast enhancement) were not included in this study. In the “case” group, we excluded the following subjects: one patient with neurotoxoplasmosis, one with Sturge-Weber Syndrome, three with tuberous sclerosis, four who were previously treated for NCC and three who had active NCC in the past. The rationale for excluding those patients was because we already know the relationship between acute symptomatic seizures and NCC during its transitional stage and by excluding patients with previous history of active NCC we aimed to minimize the influence of history of active NCC in our results (bias). From the “control group”, we excluded 13 patients who attended Headache Clinics and had also been diagnosed with epilepsy according to their medical records.

In summary, we included 119 cases and 106 controls with ages ranging from 16 to 84 years.

### Data Collection and Classification

We collected the clinical data from each patient’s medical records. We then classified cases based on their electroclinical syndrome and epilepsy etiology (Table 1) following International League Against Epilepsy’s 2010 recommendation [18]. According to this classification, we were able to define an electroclinical syndrome based on semiological data, typical electroencephalography and MRI findings. Given our primary question was to evaluate the true relationship between CNLs and MTLE-HS, we further divided cases into 2 subgroups: patients with MTLE-HS (54 subjects) and patients with all other epilepsies (65 subjects). MTLE-HS was defined as patient with the diagnosis of temporal lobe epilepsy in the presence of signs of HS on MRI scans using an epilepsy protocol.

**Table 1:** Classification of cases according to electroclinical syndromes and epilepsy etiology based on ILAE’s latest recommendation [18].

	Patients with epilepsy (n = 119)
Electroclinical Syndromes	
MTLE with hippocampal sclerosis (MTLE-HS)	54
All other syndromes except MTLE-HS	65
Unknown	25
Stroke	11
Tumor	11
Malformation of cortical development	9
Juvenile myoclonic epilepsy	4
Infection	1
Gelastic seizures with hypothalamic hamartoma	2
Epilepsy with GTCS alone	1
Trauma	1

^a^Abbreviations: ILAE = International League Against Epilepsy; MTLE-HS = mesial temporal lobe epilepsy with hippocampal sclerosis; GTCS = generalized tonic-clonic seizures.

### Brain CT Scan Analysis

We defined our primary variable of interest as the presence or absence of calcifications suggestive of NCC. We extracted information regarding the presence or absence of small parenchymal round calcifications from each radiological report. When radiological reports were not available, we assigned a qualified neurologist to evaluate the scans. According to Del Brutto’s diagnostic criteria for NCC [19], small parenchymal round calcifications are considered to be highly suggestive of NCC and constitute a major diagnostic criterion. Given that our patients also have one epidemiological criterion (NCC is endemic in Latin America) and one minor criterion (clinical manifestations suggestive of NCC such as seizures), we may classify them as probably having NCC. As CT scans were analyzed before this study, radiologists were “blinded” to our objectives and methods. The neurologist who evaluated the remaining CT scans was also blinded as to which group patients belonged.

If CNLs were present, we further classified patients according to number of CNLs (single or multiple), side of CNLs (left, right or bilateral) and location (in the temporal lobe, extratemporal and unknown). Patients with multiple calcifications were classified as located in the temporal lobe if at least one of the CNLs occurred in the temporal lobe, regardless the location of other calcifications; the unknown category was assigned if the exact location of all CNLs was not described in details on medical records (i.e. the scans were not available for review).

### MTLE-HS and Presence of CNLs

Patients with both CNLs and MTLE-HS were also divided into subgroups according to the side of occurrence of CNLs in relation to the ide of HS (bilateral, ipsilateral and contralateral).

### Statistical Analysis

We performed data analysis using SAS System (Statistical Analysis System, version 9.2, SAS Institute Inc., Cary, NC, USA) for Windows. First, we ran an exploratory analysis, measuring frequencies for categorical data and descriptive statistics for quantitative data. We compared data between cases and controls using Mann-Whitney-U tests for numerical variables and chi-square tests for categorical variables. We subsequently performed both univariate (controls versus epilepsy) and multivariate (controls versus MTLE-HS versus other epilepsies) logistic regression analysis to investigate the relationship between CNLs and MTLE-HS and between CNLs and other epilepsies. Significance was defined as p<0.05 for all analysis.

## Results

### Baseline Information

From the original sample of 268 cases and 272 controls we ultimately included 119 cases (77 women, age = 44.49 ± 13.43 years; 95%CI) and 106 controls (78 women, age = 51.27 ± 15.46 years; 95%CI). The demographic distribution of cases and controls can be seen on Table 2. Controls were found to be significantly older (p<0.001) than cases when a Mann-Whitney-U test was performed; however, both groups were similar in terms of gender distribution (p = 0.15). Detailed information for each patient and disease-controls subjects can be found in the Supporting Information **S1 Table**.

**Table 2:** Demographics of cases and controls.

	Cases	Controls	p value
**Gender**			p = 0.15 (Chi-square)
**Male**	42	28	
**Female**	77	78	
**Mean age (± SD)**	44.49 (±13.43)	51.27 (±15.46)	p<0.001 (Mann-whitney)
**Calcification**	37 (31.09%)	12 (11.32%)	p<0.001 (Chi-square)

When comparing patients with MTLE-HS and other epilepsies, we observed no difference regarding their gender distribution (68.52% women on MTLE-HS versus 61.54%; p = 0.43), but found that patients with MTLE-HS were older (48.87 years ± 12.6 versus 40.85 years ± 13.1; p<0.001).

### Comparison between Epilepsy versus Controls

We identified CNLs in 31.09% of our cases. It is of notice that 11.32% of controls also had CNLs despite the absence of symptoms suggestive of acute phase of NCC in the past, such as seizures, on their medical history. This difference was significant (p<0.001). In general, CNLs were also more frequent in women than men (27.1% versus 10%; p = 0.004). When comparing all subjects with CNLs with all those without CNLs, there was no difference regarding their age (50.86 years ± 10.83 versus 46.8 years ± 15.62; p = 0.10).

Initially, we used a binary logistic regression to compare cases and controls using presence or absence of CNLs as the dependent variable adjusted for age and gender. We found that the presence of epilepsy was associated with CNLs (OR = 5.32; 95%CI = 2.43–11.54; p<0.001), meaning that the presence of calcification increases in 5 times the chance of having epilepsy in general.

### Sub-Analysis (MTLE-HS versus Other Epilepsies versus Controls)

**Fig 2:**
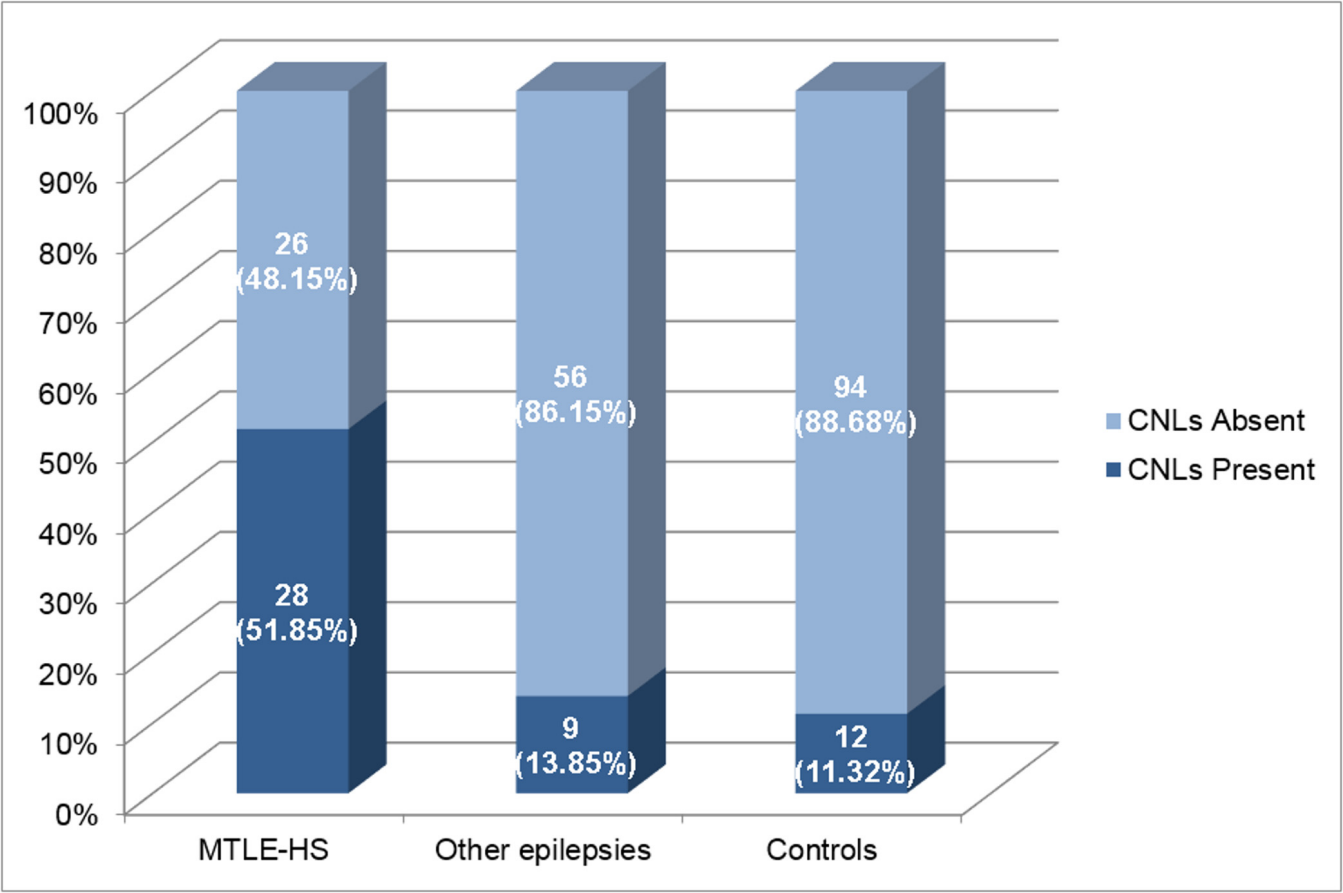
Total Number of Subjects with calcified neurocysticercotic lesions on CT scans. This graph shows the percentage of subjects with calcified neurocysticercotic lesions in each subgroup (MTLE with HS, other epilepsies, disease controls). MTLE-HS = Mesial temporal lobe epilepsy with hippocampal sclerosis, CNLs = Calcified neurocysticercotic lesions.

The sub-analysis aimed to compare patients with MTLE-HS (54 cases), controls (106 subjects) and patients with other epilepsies (65 cases). Using chi-square test on a 3x2 contingency table, we found that patients with MTLE-HS had a higher frequency of CNLs (28/54, 51.85%) when compared to both controls (12/106, 11.32%) and patients with other epilepsies (9/65, 13.85%) (p<0.001) as seen on Fig 2.

Subsequently, we performed a multinomial logistic regression using the same dependent variable (presence or absence of CNLs) with these 3 groups: MTLE-HS; other epilepsies and controls. After adjusting the analysis for age and gender, we observed that CNLs were associated with MTLE-HS compared to controls (OR = 11.27, 95%CI = 4.73–26.85; p<0.001), CNLs were associated with MTLE-HS compared to other epilepsies (OR = 5.77, 95%CI = 2.26–14.12; p<0.001)) and that CNLs were not associated with other epilepsies as compared to controls (OR = 1.95, 95%CI = 0.73–5.2; p = 0.18). For the association between CNLs and MTLE-HS the statistical power was higher than 90% (Table 3). These findings confirmed that neurocysticercotic calcifications were more frequent in patients who have MTLE-HS than in both controls and patients with other epilepsies; it also indicates that patients with CNLs were 11 times more likely to have MTLE-HS.

**Table 3:** Multinomial logistic regression model for association with CNLs.

	Odds ratio	95% CI	p value
**MTLE-HS versus controls**	11.27	4.73–26.85	<0.0001
**MTLE-HS versus other epilepsies**	5.77	2.26–14.71	0.0002
**Other epilepsies versus controls**	1.95	0.73–5.20	0.1806

### Sub-Analysis (MTLE-HS versus Location of CNLs)

**Fig 3:**
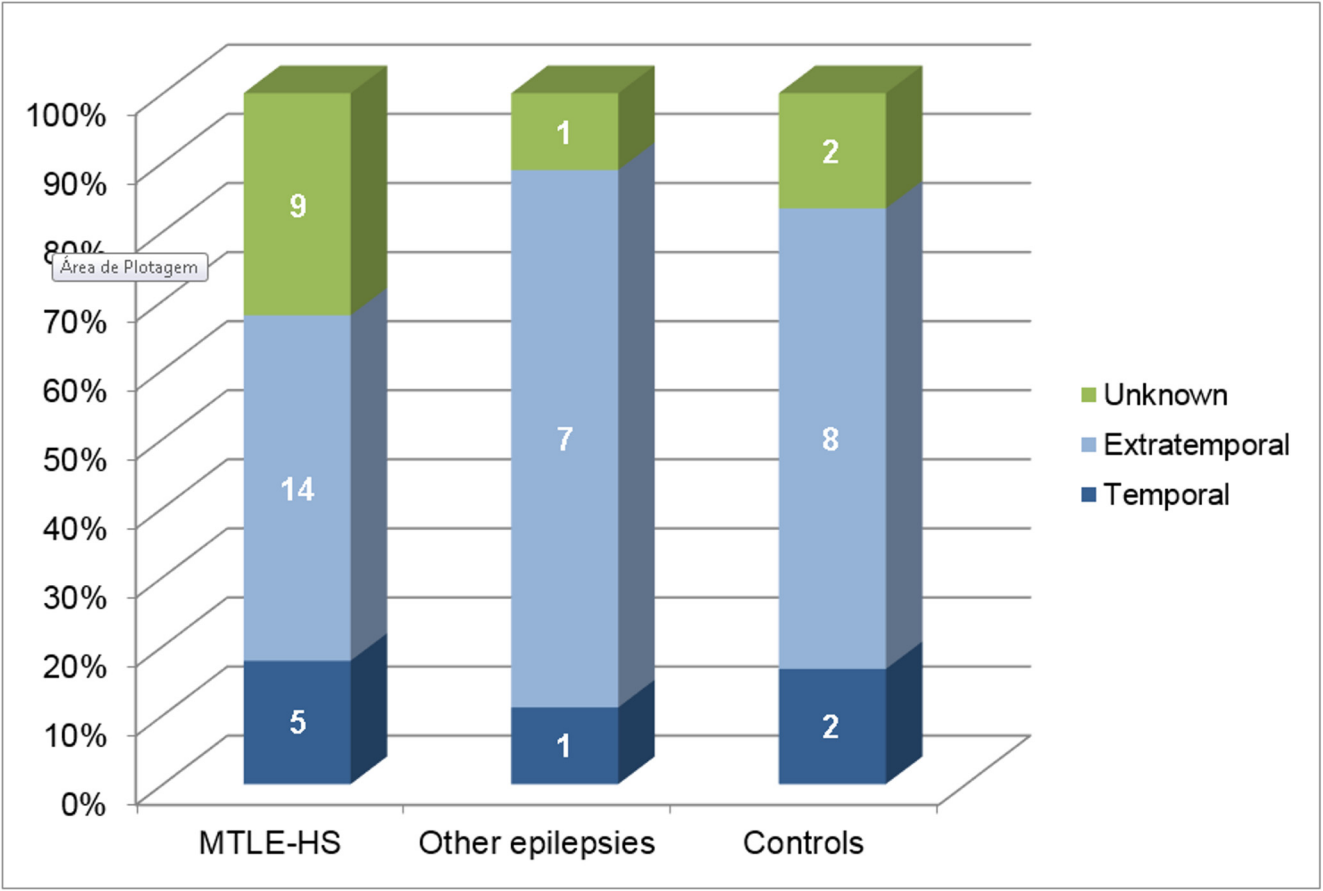
Location of neurocisticercotic calcifications in each group. This graph shows the percentage of subjects with calcifications located in the temporal lobe (Temporal), outside the temporal lobe (extratemporal) and unknown in each subgroups (MTLE-HS, other epilepsies, disease controls). There were no significant group differences considering the presence of calcifications in the temporal lobes. (p = 0.58) MTLE-HS = Mesial temporal lobe epilepsy with hippocampal sclerosis.

In this sub-analysis, we included only cases and controls with CNLs and divided them in 3 groups: MTLE-HS (n = 28), other epilepsies (n = 9) and controls (n = 12). We analyzed whether there were any differences in location of CNLs (temporal, extratemporal, unknown) amongst those 3 groups. We did not find a significant difference (p = 0.58) in CNLs location between them as seen in Fig 3.

**Fig 4:**
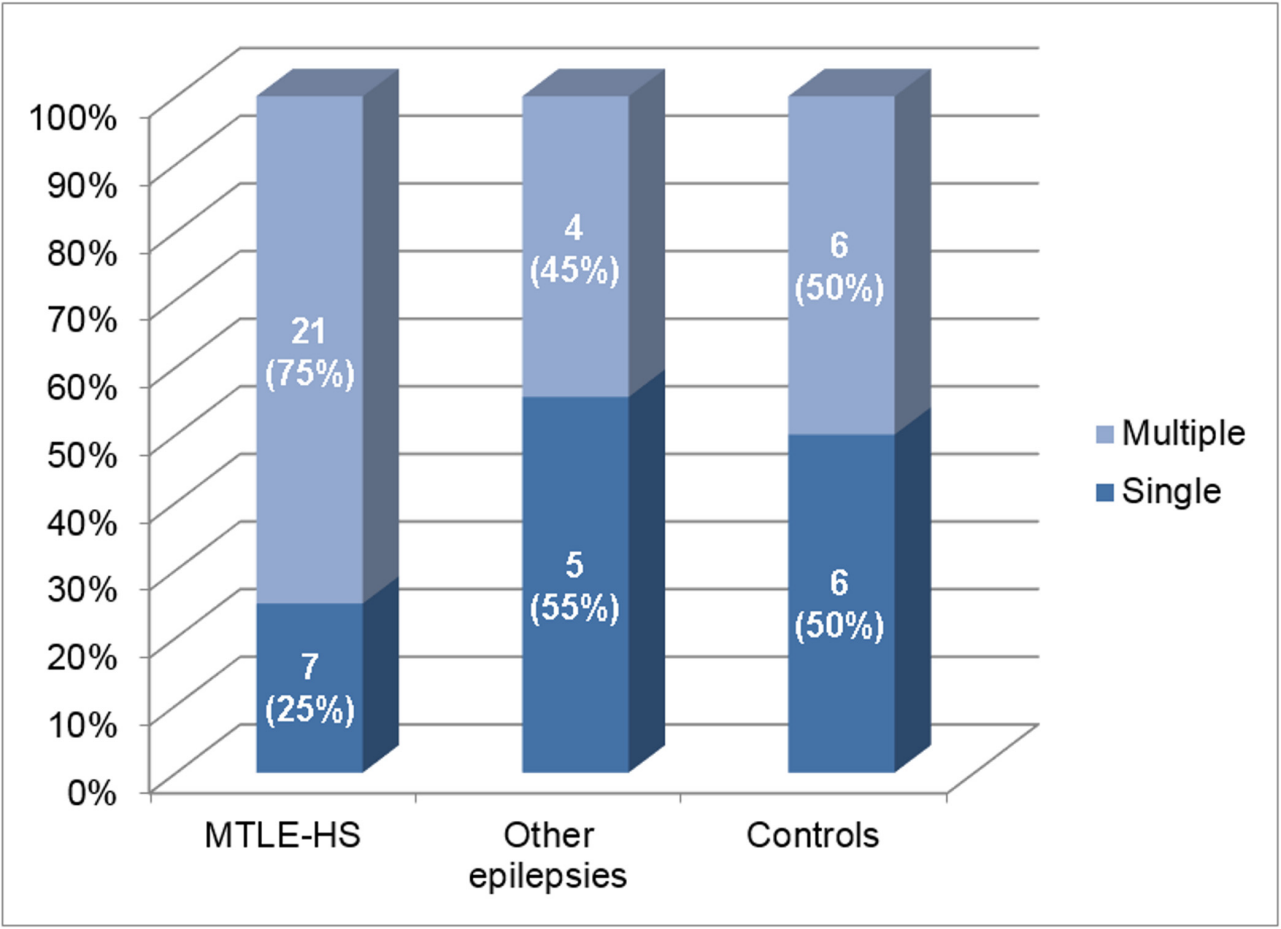
Number of neurocisticercotic calcifications in each group. This graph shows the percentage of subjects with single or multiple calcifications in each subgroup (MTLE-HS, other epilepsies, disease controls). There were no significant group differences regarding the load of lesions (p = 0.14). MTLE-HS = Mesial temporal lobe epilepsy with hippocampal sclerosis.

We did not find differences in CNLs load between these 3 groups (p = 0.14), as illustrated in Fig 4. The analysis of the CNLs side comparing to the side of HS showed that 21% of the CNLs were ipsilateral to the side HS, 17% were contralateral to the HS side and 62% had bilateral CNLs (including in this last group one patient that had bilateral HS).

## Discussion

We found an association between neurocysticercotic calcifications and MTLE-HS. Our data showed that the association between CNLs and epilepsy in general is much influenced by patients with MTLE-HS (45.4% in our cohort); since the exclusion of MTLE-HS from the model/analysis yielded non-significant association between CNLs and other types of epilepsy.

The frequency of CNLs in our study is seemingly high (31% for cases overall, and 11% for disease controls). However, the frequency of CNLs in patients with epilepsy has been high in studies from the same region [2]. In addition, a recent study that invited 248 volunteers (age > 60 years) to perform CT or MRI scans also detected CNLs in 11% of these individuals (only 4 of these had epilepsy) [14].

Our findings are similar to that of Velasco et al [2], showing an association between CNLs and HS and suggesting that NCC is possibly a form of initial precipitating injury (IPI), rather than a major cause of epilepsy. The main difference between our study and that of Velasco et al [2] and others [14, 20, 21], is the inclusion of a disease control group, thus, providing stronger evidence for these associations, at least in the context of a hospital based population.

We also found a higher frequency of CNLs amongst women, as seen in previous studies [22, 23, 24]. The possible cause of this finding is still unknown.

We discuss here four possible explanations for our findings, as suggested previously in the literature [13, 15]. The first would be coincidence. The second possible explanation would be that NCC and MTLE-HS are associated, but are not causally related (e.g. due to a third unrelated factor, such as poor socioeconomic background). Third, NCC may be a direct cause of MTLE-HS or epilepsy. Fourth, the association between NCC and MTLE-HS is as part of the epileptogenic process (trigger) but not a direct causative factor.

The first theory claims that since both diseases are relatively common entities in endemic areas, they just happen to appear in the same patient as a coincidence. We do recognize that in some patients that might be the case, but we believe that this theory seems unable to explain the very high frequency of CNLs in patients with MTLE-HS found in our study (51.85%) or in other studies: 17.7% and 37.2% in two studies of MTLE-HS patients submitted to surgery [10, 25] or 35.6% in a retrospective cohort [26].

The second theory does not deny a correlation between NCC and MTLE-HS, but it claims that there is no causative association between both: a third variable would be implicated in the pathophysiology of both. In statistics, correlation does not mean causation. In the case of NCC and MTLE-HS, for example, low economic status or genetic susceptibility to both conditions could lead to this association [15]. Although both groups (cases and controls) in our study had the same socioeconomic background, we cannot rule out the possibility of poverty or genetic susceptibility being the third variable to explain both NCC and MTLE-HS in our series.

The third theory states that MTLE-HS may be caused by direct inflammation and structural damage to the hippocampi or cortex by the acute phase of cysticerci located in the vicinity. This might be true for some specific cases, but probably does not explain all of them. For example, there are case reports of patients with a cysticercus in the hippocampus with MTLE-HS [27, 28]. Thus, we do not think this is the most important mechanism by which NCC could cause MTLE-HS, because in patients with MTLE-HS and NCC the calcified lesions are not necessarily located in the temporal lobes, as we demonstrated earlier in a different series of patients in our center [29]. In that study [29], like in the present study, we found no relationship between localization of CNLs (temporal or extra-temporal) and seizure semiology (MTLE or other types of epilepsy).

The fourth theory suggests the presence of NCC could work as an IPI that would lead to HS, via multiple mechanisms, and then HS itself would cause chronic seizures, and frequently refractory epilepsy. There is evidence that some specific kinds of lesions or aggressions may cause mesial temporal lobe damage and as a consequence lead to HS, which would then cause MTLE [30, 31]. These aggressions are known as IPIs [31]. The most studied IPIs are prolonged febrile seizures, neonatal hypoxia, status epilepticus, trauma and meningoencephalitis [32]. This theory suggests that one plausible explanation for the association we have found is that NCC most likely represents one IPI in patients with predisposition to have seizures and therefore to develop MTLE-HS. Clinical or subclinical inflammation on the acute phase of infection would damage or aggravate a previously injured or predisposed hippocampus. The presence of inflammation is widely accepted [33] and a MRI perfusion study [1] suggests that even after resolution of the acute phase, with the formation of CNLs, there may still be inflammation induced by the acute phase. The CNLs would function both as a marker of previous damage to the hippocampus and as an active injury [34]. Another possible mechanism of epileptogenesis, according to some authors, would be the degenerating cysts leading to the development of electrical activity in the hippocampi through the phenomena of kindling, interictal discharge or even subclinical seizures [13]. A previous study comparing patients with MTLE-HS with or without CNLs found no difference in their post-surgical prognosis [10], which in our opinion (although not directly proven by the present study) further supports the notion of NCC as an IPI, given the presence of CNLs after surgery (even in the hemisphere contralateral to the surgical resection) would not influence post-surgical outcome. This goes along with our finding that NCC is more associated with HS, and hence MTLE-HS, than with other epilepsies in the context of our series. Another circumstantial evidence that NCC may be an IPI for MTLE-HS is the higher prevalence of MTLE-HS in developing countries, where NCC is endemic, compared to developed countries [35, 36].

### Limitations

There are some limitations to our study: our controls were not healthy volunteers, as it would be unethical to submit healthy controls to unneeded radiation exposure. Therefore, it is possible that the frequency of CNLs differs between a healthy population and controls selected from a Headache Clinics [37]. However, as discussed above, the rate of CNLs in our disease controls was similar to that reported by Dell Bruto et al. [14].

Another limitation is that 43 out of 106 controls were using antiepileptic drugs, which raises the possibility that some of them did not present with seizures simply because they were already being treated.

Another limitation was that all patients with CNLs were considered to have NCC despite not fulfilling criteria for definitive diagnosis. According to the most recent diagnostic criteria [19], those patients would be considered to have a probable diagnosis of NCC based on the findings of highly suggestive lesions on CT scan plus suggestive clinical manifestations and living in endemic area. Additionally, our study is retrospective and uses data that had already been described in medical charts or radiological reports. Another limitation is that not all controls had undergone MRI scans, which might have shown HS even in asymptomatic (e.g. without epilepsy) patients [38]. In addition, 45.8% of 119 cases had MTLE-HS probably due to a referral bias, as our institution is a reference center in its region for the treatment of refractory epilepsy. That said, it might not be feasible to prove or disprove the association between CNLs and MTLE-HS in a population-based study, given the relatively low prevalence of the former in the overall population.

In conclusion, our study showed a significant association between CNLs and MTLE-HS and indirectly suggests that NCC may acts as an IPI in patients with predisposition to develop HS or to have seizures. However, our data does not support the association between NCC and other types of epilepsy. It is important to note that these findings cannot be extrapolated to the general population, first, because it is a series from a tertiary center, and second, because of the heterogeneity of the other subgroups in our study. In this article, we review several different mechanisms by which NCC and MTLE-HS might be associated. We believe that the relationship between NCC and MTLE-HS is complex and probably results from the combination of all possible mechanisms discussed herein.

Given the controversy and lack of strong statistical evidences addressing the relationship between CNLs, epilepsy and HS the results reported herein provide important statistical evidence regarding the risk of epilepsy in the presence of CNLs. We found that the presence of CNLs increases in 11 times the likelihood of having epilepsy with HS. This finding is important for the understanding of the pathophysiology of NCC and HS and the concept of dual pathology. The decision of considering the presence of CNLs in a patient with MTLE-HS as a possible IPI, and not as a dual pathology, may have implications in treatment and prognosis for patients with CNLs and HS.

### Statistical Analysis

Statistical analysis was conducted by Dr. Morita (Department of Neurology), Dr. Cendes (Department of Neurology) and Biostatistical Support Group from FCM—University of Campinas.

## Supporting Information

S1 Table Clinical and demographic data for all patients and disease-controls. (XLSX)

## References

[1] Gupta RK, Awasthi R, Rathore RK, Verma A, Sahoo P, Paliwal VK, et al (2012) Understanding epileptogenesis in calcified neurocysticercosis with perfusion MRI. Neurology 78:618–625. 10.1212/WNL.0b013e318248deae 22302547

[2] Velasco TR, Zanello PA, Dalmagro CL, Araújo D Jr, Santos AC, Bianchin MM, et al (2006) Calcified cysticercotic lesions and intractable epilepsy: a cross sectional study of 512 patients. Journal of neurology, neurosurgery, and psychiatry 77: 485–488. 16543527 10.1136/jnnp.2005.078675 PMC2077509

[3] Montano SM, Villaran MV, Ylquimiche L, Figueroa JJ, Rodriguez S, Bautista CT, et al (2005) Neurocysticercosis: association between seizures, serology, and brain CT in rural Peru. Neurology 65:229–233. 16043791 10.1212/01.wnl.0000168828.83461.09

[4] Rajshekhar V1, Raghava MV, Prabhakaran V, Oommen A, Muliyil J. (2006) Active epilepsy as an index of burden of neurocysticercosis in Vellore district, India. Neurology 67: 2135–2139. 17190933 10.1212/01.wnl.0000249113.11824.64

[5] de Souza A, Nalini A, Kovoor JM, Yeshraj G, Siddalingaiah HS, Thennarasu K. (2010) Natural history of solitary cerebral cysticercosis on serial magnetic resonance imaging and the effect of albendazole therapy on its evolution. J Neurol Sci 288: 135–141. 10.1016/j.jns.2009.09.018 19875133

[6] Carpio A, Escobar A, Hauser WA. (1998) Cysticercosis and epilepsy: a critical review. Epilepsia 39: 1025–1040. 9776322 10.1111/j.1528-1157.1998.tb01287.x

[7] Singh G, Sachdev MS, Tirath A, Gupta AK, Avasthi G. (2000) Focal cortical-subcortical calcifications (FCSCs) and epilepsy in the Indian subcontinent. Epilepsia 41:718–726. 10840405 10.1111/j.1528-1157.2000.tb00234.x

[8] Nash TE, Del Brutto OH, Butman JA, Corona T, Delgado-Escueta A, Duron RM, et al (2004) Calcific neurocysticercosis and epileptogenesis. Neurology 62: 1934–1938. 15184592 10.1212/01.wnl.0000129481.12067.06 PMC2912520

[9] Nash TE, Pretell EJ, Lescano AG, Bustos JA, Gilman RH, Gonzalez AE, et al (2008) Perilesional brain oedema and seizure activity in patients with calcified neurocysticercosis: a prospective cohort and nested case-control study. Lancet Neurol. 7: 1099–1105. 10.1016/S1474-4422(08)70243-6 18986841 PMC3725597

[10] Leite JP, Terra-Bustamante VC, Fernandes RM, Santos AC, Chimelli L, Sakamoto AC, et al (2000) Calcified neurocysticercotic lesions and postsurgery seizure control in temporal lobe epilepsy. Neurology 55:1485–1491. 11094102 10.1212/wnl.55.10.1485

[11] Engel JJ. (2001) Mesial temporal lobe epilepsy: what have we learned? Neuroscientist 7: 340–352. 11488399 10.1177/107385840100700410

[12] Wieser HG, Epilepsy ICoNo. (2004) ILAE Commission Report. Mesial temporal lobe epilepsy with hippocampal sclerosis. Epilepsia 45: 695–714. 15144438 10.1111/j.0013-9580.2004.09004.x

[13] Singla M, Singh P, Kaushal S, Bansal R, Singh G. (2007) Hippocampal sclerosis in association with neurocysticercosis. Epileptic Disord 9: 292–299. 17884753 10.1684/epd.2007.0122

[14] Del Brutto OH, Salgado P, Lama J, Del Brutto VJ, Campos X, Zambrano M et al (2015) Calcified Neurocysticercosis Associates with Hippocampal Atrophy: A Population-Based Study. Am J Trop Med Hyg 92:64–8. 10.4269/ajtmh.14-0453 25349375 PMC4347393

[15] Bianchin MM, Velasco TR, Santos AC, Sakamoto AC. (2012) On the relationship between neurocysticercosis and mesial temporal lobe epilepsy associated with hippocampal sclerosis: coincidence or a pathogenic relationship? Pathog Glob Health 106: 280–285. 10.1179/2047773212Y.0000000027 23265552 PMC4005111

[16] Fisher RS, Acevedo C, Arzimanoglou A, Bogacz A, Cross H, Elger CE, et al A Practical Clinical definition of epilepsy. (2014) Epilepsia 55: 475–482. 10.1111/epi.12550 24730690

[17] Garcia HH, Del Brutto OH. (2003) Imaging findings in neurocysticercosis. Acta Trop 87: 71–78. 12781380 10.1016/s0001-706x(03)00057-3

[18] Berg AT, Berkovic SF, Brodie MJ, Buchhalter J, Cross JH, van Emde Boas W, et al (2010) Revised terminology and concepts for organization of seizures and epilepsies: Report of the ILAE Commission on Classification and Terminology, 2005–2009. Epilepsia 51: 676–685. 10.1111/j.1528-1167.2010.02522.x 20196795

[19] Del Brutto OH. (2012) Diagnostic criteria for neurocysticercosis, revisited. Pathog Glob Health 106: 299–304. 10.1179/2047773212Y.0000000025 23265554 PMC4005113

[20] Garcia-Noval J, Moreno E, de Mata F, Soto de Alfaro H, Fletes C, Craig PS, et al (2001) An epidemiological study of epilepsy and epileptic seizures in two rural Guatemalan communities. Ann Trop Med Parasitol 95: 167–175. 11299123 10.1080/00034980120050260

[21] Oliveira MC, Martin MG, Tsunemi MH, Vieira G, Castro LH. (2014) Small calcified lesions suggestive of neurocysticercosis are associated with mesial temporal sclerosis. Arq Neuropsiquiatr. 72: 510–6. 25054983 10.1590/0004-282x20140080

[22] Bianchin MM, Velasco TR, Takayanagui OM, Sakamoto AC. (2006) Neurocysticercosis, mesial temporal lobe epilepsy, and hippocampal sclerosis: an association largely ignored. Lancet Neurol 5: 20–21. 16361019 10.1016/S1474-4422(05)70269-6

[23] Bianchin MM, Velasco TR, Coimbra ER, Gargaro AC, Escorsi-Rosset SR, Wichert-Ana L, et al. (2013) Cognitive and surgical outcome in mesial temporal lobe epilepsy associated with hippocampal sclerosis plus neurocysticercosis: a cohort study. PLoS One 10.1371/journal.pone.006094 PMC3632568 23613762

[24] Bianchin M, Velasco T, Araújo D, Wichert-Ana L, Alexandre V Jr, Terra-Bustamante VC, et al (2008) Chronic neurocysticercosis is anatomically related with hippocampal sclerosis in refractory mesial temporal lobe epilepsy plus neurocysticercosis. Epilepsia 49(suppl 7): S487.

[25] Bianchin MM, Velasco TR, Wichert-Ana L, Alexandre V Jr, Terra-Bustamante VC, Inuzuka LM, et al (2005) In endemic areas, neurocysticercosis is highly prevalent in patients with mesial temporal lobe epilepsy, but it is not a risk factor for poor surgical outcome or post-surgical cognitive decline. Epilepsia 46(suppl 8): S236.

[26] Bianchin MM, Velasco TR, Araújo D Jr, Alexandre V Jr, Wichert-Ana L, Terra-Bustamante VC, et al (2006) Clinical and Electrophysiological Differences between Mesial Temporal Lobe Epilepsy and Mesial Temporal Lobe Epilepsy Plus Neurocysticercosis. Epilepsia 47(suppl 4): S244–S245.

[27] da Silva AV, Martins HH, Marques CM, Yacubian EM, Sakamoto AC, Carrete H Jr, et al (2006) Neurocysticercosis and microscopic hippocampal dysplasia in a patient with refractory mesial temporal lobe epilepsy. Arq Neuropsiquiatr 64: 309–313. 16791376 10.1590/s0004-282x2006000200026

[28] Kobayashi E, Guerreiro CA, Cendes F. (2001) Late onset temporal lobe epilepsy with MRI evidence of mesial temporal sclerosis following acute neurocysticercosis:case report. Arq Neuropsiquiatr 59: 255–258. 11400037 10.1590/s0004-282x2001000200021

[29] da Gama CN, Kobayashi E, Li LM, Cendes F. (2005) Hippocampal atrophy and neurocysticercosis calcifications. Seizure 14: 85–8. 15694560 10.1016/j.seizure.2004.10.005

[30] Cendes F. (2005) Progressive hippocampal and extrahippocampal atrophy in drug resistant epilepsy. Curr Opin Neurol 18: 173–177. 15791149 10.1097/01.wco.0000162860.49842.90

[31] Meyer A, Falconer MA, Beck E. (1954) Pathological findings in temporal lobe epilepsy. J Neurol Neurosurg Psychiatry 17: 276–285. 13212417 10.1136/jnnp.17.4.276 PMC503198

[32] Lewis DV. (2005) Losing neurons: selective vulnerability and mesial temporal sclerosis. Epilepsia 46(suppl 7): S39–44. 10.1111/j.1528-1167.2005.00306.x 16201994

[33] Garcia HH, Del Brutto OH. (2005) Neurocysticercosis: updated concepts about an old disease. Lancet Neurol 4: 653–661. 16168934 10.1016/S1474-4422(05)70194-0

[34] Rathore C, Thomas B, Kesavadas C, Abraham M, Radhakrishnan K. (2013) Calcified neurocysticercosis lesions and antiepileptic drug-resistant epilepsy: a surgically remediable syndrome? Epilepsia 54: 1815–1822. 10.1111/epi.12349 24032594

[35] Ngugi AK, Kariuki SM, Bottomley C, Kleinschmidt I, Sander JW, Newton CR, et al (2011) Incidence of epilepsy: a systematic review and meta-analysis. Neurology 77: 1005–1012. 10.1212/WNL.0b013e31822cfc90 21893672 PMC3171955

[36] Sander JW. (2003) The epidemiology of epilepsy revisited. Curr Opin Neurol 16: 165–170. 12644744 10.1097/01.wco.0000063766.15877.8e

[37] Agapejev S. (2003) Clinical and epidemiological aspects of neurocysticercosis in Brazil: a critical approach. Arq Neuropsiquiatr 61: 822–828. 14595490 10.1590/s0004-282x2003000500022

[38] Kobayashi E, Li LM, Lopes-Cendes I, Cendes F. (2002) Magnetic resonance imaging evidence of hippocampal sclerosis in asymptomatic, first-degree relatives of patients with familial mesial temporal lobe epilepsy. Arch Neurol 59: 1891–1894. 12470176 10.1001/archneur.59.12.1891

